# Supplementary material for: Optimising risk-based surveillance for early detection of invasive plant pathogens
Source: PLoS Biol. 2020 Oct 12;18(10):e3000863. doi: 10.1371/journal.pbio.3000863 (PMC7581011; doi:10.1371/journal.pbio.3000863)
Supplement: S1 Text — (DOCX) [file pbio.3000863.s001.docx]

**Optimising risk-based surveillance for early detection of invasive plant pathogens: S1 Text**

**Model formulation**

The model is a spatially-explicit susceptible-infected model, which tracks – in continuous time – the infection status of each 1km x 1km cell. The model accounts for heterogeneous densities of citrus and rates of pathogen entry, as well as local bulking-up of pathogen densities at any location and dispersal between cells.

The model runs on a rectangular grid of 1km^2^ cells, of extent 672 x 732, to bound the whole of mainland Florida (i.e. excluding the Florida Keys) – encompassing a total of 148,089 cells. The model takes two spatially-heterogeneous inputs, which specify – for each location $L$ – the following (fixed) quantities:

- $\rho_{L}$= proportion of the cell $L$ containing citrus;

- $\varepsilon_{L}$= rate of pathogen entry into cell $L$.

Only the 35,157 cells across the landscape with $\rho_{L}\geq0.0025$ (i.e. which contain at least 2,500m^2^ of citrus) are tracked. This set of active cells is denoted as $A$. Filtering out cells containing only very small densities of host plants led to a significant increase in computational speed with minimal impact on the spatiotemporal pattern of simulated pathogen spread, due to the low probability of infection in and further spread from these cells even if included. Although this feature of the model fragments the landscape, spread within the main commercial citrus growing area in the centre of the Floridian peninsula remains well connected, and long-range spread is still captured through repeated pathogen incursions.

Cells are partitioned into two disjoint sets: infected $\left( I \right)$ and susceptible$\left( S \right)$, with $I\cup S=A$. Susceptible cells are selected to receive infection from outside the study area randomly, with the rate of pathogen entry calculated from the product of a global rate parameter, the cell’s individual relative primary infection rate, and the citrus density. We assume the first infection of a cell leads to $\sigma_{0}=0.006$ of the citrus within it becoming infected, and that thereafter the pathogen bulks-up logistically at rate $r$*.* Following first infection, within-cell spread of the pathogen is assumed to be dominated by local spread, which occurs deterministically[1]. This simplifies many of the calculations and allows large numbers of simulations to be run in a shorter time period - which is a large advantage of our current model. In particular, we assume that if cell $L\in I$ is first infected at $t_{L}$, at any subsequent time the density of infected host is given by:

$$\omega_{L}\left( t \right)=\frac{\rho_{L}}{1+\left( \frac{1}{\sigma_{0}}-1 \right)e^{-r\left( t-t_{L} \right)}}$$

The only stochastic transitions tracked by our model are pathogen spread into cells from outside the landscape (‘primary infection’) and between cells in the landscape (‘secondary infection’). The time-dependent net rate at which any uninfected cell $L\in S$ becomes infected, $\lambda_{L}\left( t \right)$, is:

$$\lambda_{L}\left( t \right)=\rho_{L}\left( \varepsilon_{L}+\beta\sum_{\bar{L}\in I,\bar{L}\neq L} K\left( L,\bar{L} \right)\omega_{\bar{L}}\left( t \right) \right)$$

in which $\beta$ scales the secondary infection rate and $K\left( L,\bar{L} \right)$ is a dispersal kernel linking uninfected cell $L$ with infected cell $\bar{L}$. We use an appropriately normalised two-dimensional exponential kernel, where:

$K\left( L,\bar{L} \right)=\frac{1}{2\pi\alpha^{2}}\exp\left( \frac{-d_{L,\bar{L}}}{\alpha} \right)$

Here, $\alpha$ is a dispersal scale parameter and $d_{L,\bar{L}}$ is the distance between the centres of cells $L$ and $\bar{L}$. The process of infection is simulated according to an appropriate update to Gillespie algorithm[2,3] which accounts for time-inhomogeneous rates.

We modelled the increase in detectability $\left( \varphi\right)$ in each cell over time using the same approach described for infectiousness above, with a rate parameter of $s$ and an initial detectability of $\varsigma_{0}$.

$$\varphi_{L}\left( t \right)=\frac{1}{1+\left( \frac{1}{\varsigma_{0}}-1 \right)e^{-s\left( t-t_{L} \right)}}$$

We fit the infection rate, $\beta$, the scale of dispersal $\alpha$, the rates of within-cell bulk up and detectability increase, $r$ and $s$, and the initial cell infectiousness and detectability,$\sigma_{0}$ and $\varsigma_{0}$, to spread data as described below.

**Optimisation algorithm**

Our algorithm first randomly selects the required number of sites and calculates the mean probability of detection, $p\left( \Omega,n,\Delta t \right)$, for the given sampling arrangement $\Omega_{j}$ (under the given surveillance parameters, $n,\Delta t$), as described in the main text. We used this detection probability as the ‘objective function’ in the optimisation algorithm, which needs to be maximised. For a prespecified number of iterations $\left( J \right)$, the algorithm proceeds by sequentially replacing a single site with another randomly selected one before calculating the objective function again. The arrangement with the new site is then either accepted or rejected before another site is randomly replaced and the process repeated. Each time, the following Metropolis criterion is used to estimate the probability of accepting the new site:

$$P\left( \Omega_{j} \to\Omega_{j+1} \right)=1 \mathrm{if} p\left( \Omega_{j+1},n,\Delta t \right)>p\left( \Omega_{j},n,\Delta t \right)$$

$$P\left( \Omega_{j} \to\Omega_{j+1} \right)=\exp\left( \frac{p\left( \Omega_{j+1},n,\Delta t \right)-p\left( \Omega_{j},n,\Delta t \right)}{{temp}_{j}} \right) \mathrm{if} p\left( \Omega_{j+1},n,\Delta t \right)<p\left( \Omega_{j},n,\Delta t \right)$$

If the objective function was equal between iterations, then the new arrangement was accepted with a probability of 0.5. The ‘temperature’ of the algorithm $\left( temp \right)$ is multiplied by the cooling rate $\left( alpha \right)$ at the end of each iteration (i.e. an exponential cooling schedule[4]). The result of this is that in the early stages of the algorithm, $temp$ is high and so is the probability of accepting ‘worse’ arrangements of sampling sites - thereby encouraging a full exploration of the full parameter space, avoiding any local maxima. As the algorithm progresses, $temp$ decreases and it becomes increasingly likely that worse arrangements are rejected (although there initially remains some freedom to explore the parameter space). In the late stages of the algorithm, all arrangements which give a lower probability of detection are rejected, allowing a good approximation of the true optimal arrangement to be found.

**Model Parametrisation**

The citrus density was estimated by summing 1km square gridded data on the distribution of residential and commercial citrus in the state as described in the main text. Following introduction, we assume that the prevalence of infection (and therefore, the infectiousness of the cell) grows logistically. We also assume that the detectability of infection increases logistically, although not necessarily at the same rate. To determine the rate of increase in detectability, we fitted a logistic model to data on the progression of visually detectable infection over time, collected in the ‘Devil’s Garden’ plantation in southern Florida[5,6]. This gave a growth rate of about 0.0028 per day, which is about 1.0 per year. Using data on the development of symptoms over time (described below), and assuming a lag of around six months between infection and first expression of symptoms[7], we assumed that the rate of logistic growth in the prevalence was 1.25 per year. The diagnostic sensitivity of visual inspection was assumed to be 0.5 (inferred from a comparison of PCR and visual inspection for detection of infection in Floridian citrus groves[8].

In order to estimate the rate and pattern of secondary spread, we first assumed that the mean distance of spread (in two dimensions) was around 20km, which corresponds to data obtained in a recent study[9]. We then ran ten simulations of Las spread with identical parameters, restricting initial entry to the four counties found to be infected at the time of first detection (Miami-Dade, Broward, Palm Beach and Martin, in the south east of the state)[10], and assuming a low rate of pathogen entry (0.05), following the distribution predicted by the travel census model. We repeated this process whilst incrementally increasing the rate of secondary spread until the prevalence after ten years exceeded 95%.

**References**

1. Klepac P, Kissler S, Gog J. Contagion! The BBC Four Pandemic – The model behind the documentary. Epidemics. 2018;24: 49–59. doi:10.1016/j.epidem.2018.03.003

2. Gillespie DT. A general method for numerically simulating the stochastic time evolution of coupled chemical reactions. J Comput Phys. 1976;22: 403–434. doi:10.1016/0021-9991(76)90041-3

3. Gillespie DT. Exact stochastic simulation of coupled chemical reactions. J Phys Chem. 1977;81: 2340–2361. doi:10.1021/j100540a008

4. Kirkpatrick S, Gelatt CD Jr, Vecchi MP. Optimization by simulated annealing. Science. 1983;220: 671–680. doi:10.1126/science.220.4598.671

5. Sankaran S, Mishra A, Maja JM, Ehsani R. Visible-near infrared spectroscopy for detection of huanglongbing in citrus orchards. Comput Electron Agric. 2011;77: 127–134. doi:10.1016/j.compag.2011.03.004

6. Gast T, Irey MS, Hou H. A database for analysis of *Diaphorina citri* population monitoring data from commercial groves. Proceedings of the 2nd International Research Conference on Huanglongbing. 2011.

7. Gottwald TR. Current epidemiological understanding of citrus huanglongbing. Annu Rev Phytopathol. 2010;48: 119–139. doi:10.1146/annurev-phyto-073009-114418

8. Irey MS, Gast T, Gottwald TR. Comparison of visual assessment and polymerase chain reaction assay testing to estimate the incidence of the huanglongbing pathogen in commercial Florida citrus. Proc Fla State Hortic Soc. 2006;119: 89–93.

9. Parnell S, Gottwald TR, Riley T, van den Bosch F. A generic risk-based surveying method for invading plant pathogens. Ecol Appl. 2014;24: 779–790. doi: 10.1890/13-0704.1

10. Halbert SE. The discovery of huanglongbing in Florida. Proceedings of the 2nd International Citrus Canker and Huanglongbing Research Workshop. 2005.
