# Supplementary figures and images for: Optimising risk-based surveillance for early detection of invasive plant pathogens
Source: PLoS Biol. 2020 Oct 12;18(10):e3000863. doi: 10.1371/journal.pbio.3000863 (PMC7581011; doi:10.1371/journal.pbio.3000863)

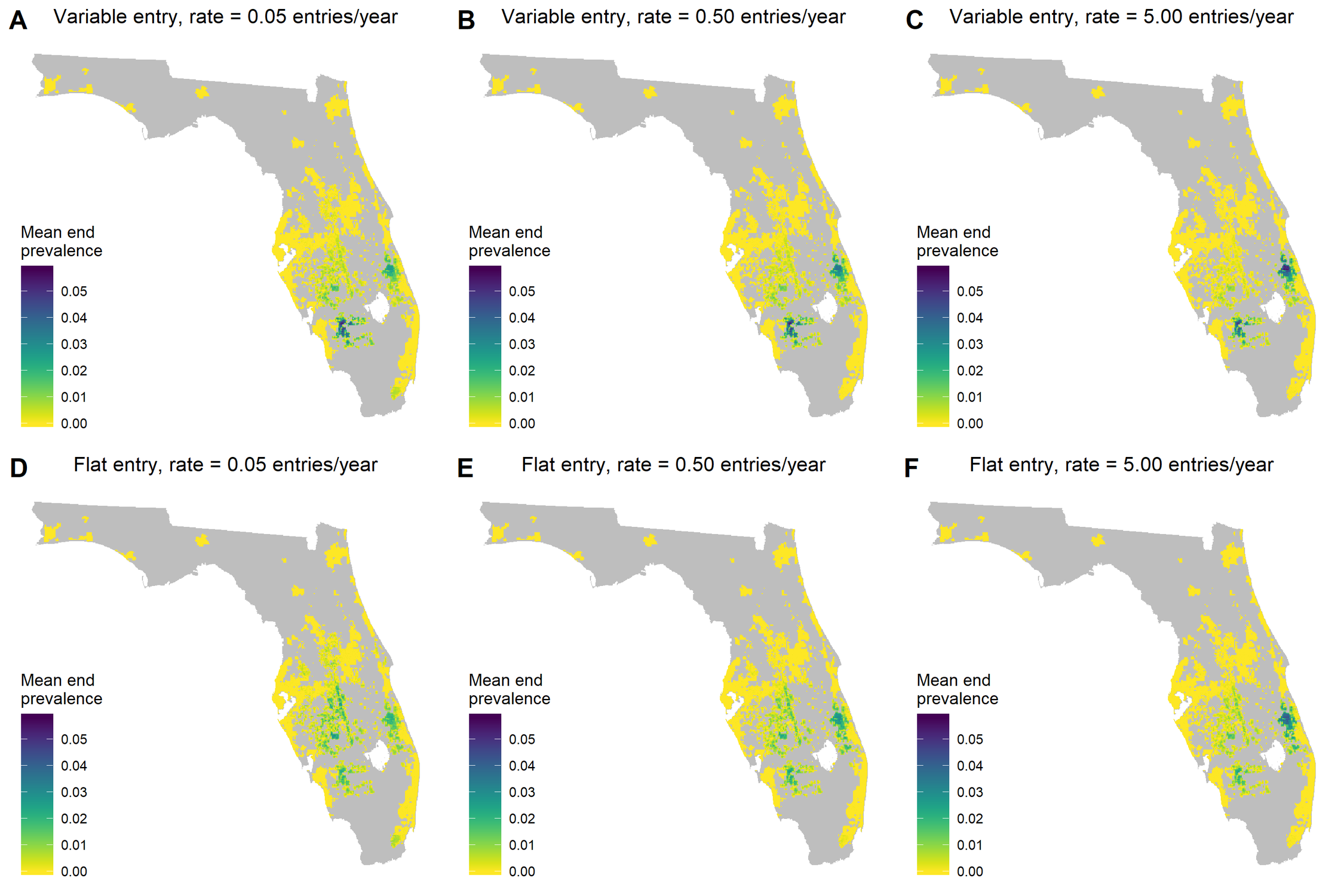

Supplement: S1 Fig — These plots demonstrate the impact of varying the characteristics of pathogen entry into the state on the mean prevalence at the point the state-wide prevalence threshold of 1% is reached. Plots A–C show the mean prevalence when the probability of pathogen entry into any given site is affected by the density of citrus host and the travel census probabilities, and plots D–F show the mean prevalence when only the citrus density influences the probability of pathogen entry. Plots A and D show a ‘low’ mean rate of pathogen entry up to 0.05 entries per year, B and E show a ‘medium’ mean rate of up to 0.5 entries per year, and C and F show a ‘high’ rate of up to 5 entries per year. Higher rates of entry result in more variability in end prevalence estimates throughout the state. The data used to create these plots can be found at https://doi.org/10.17866/rd.salford.12759929.v1 (file ‘spatialData_primaryInf.csv’). (TIF) [file pbio.3000863.s002.tif]

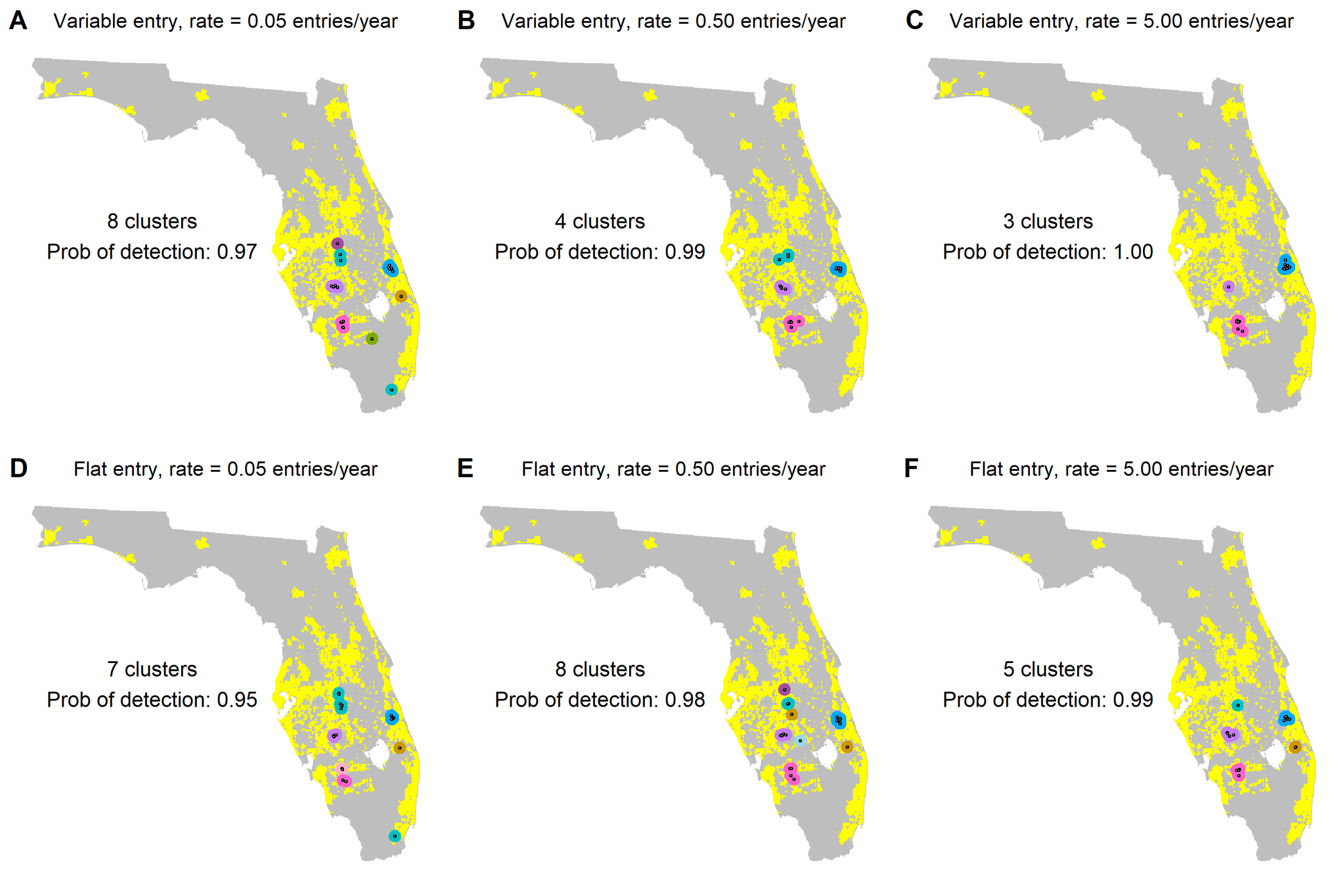

Supplement: S2 Fig — These plots show the spatial arrangement of optimal sites (taken from a single optimisation run) and clusters of these sites, along with the detection probability, when the rate of pathogen entry is varied. Plots A–C show the distribution when the probability of pathogen entry into any given site is affected by the density of citrus host and the travel census probabilities. Plots D–F show the distribution when only the citrus density influences the probability of pathogen entry. Plots A and D show a ‘low’ mean rate of pathogen entry up to 0.05 entries per year, B and E show a ‘medium’ mean rate of up to 0.5 entries per year, and C and F show a ‘high’ rate of up to 5 entries per year. Estimates of the number of clusters and the probability of detection under the different sampling patterns are also shown. The data used to create these plots can be found at https://doi.org/10.17866/rd.salford.12759929.v1 (file ‘spatialData_primaryInf.csv’). (TIF) [file pbio.3000863.s003.tif]

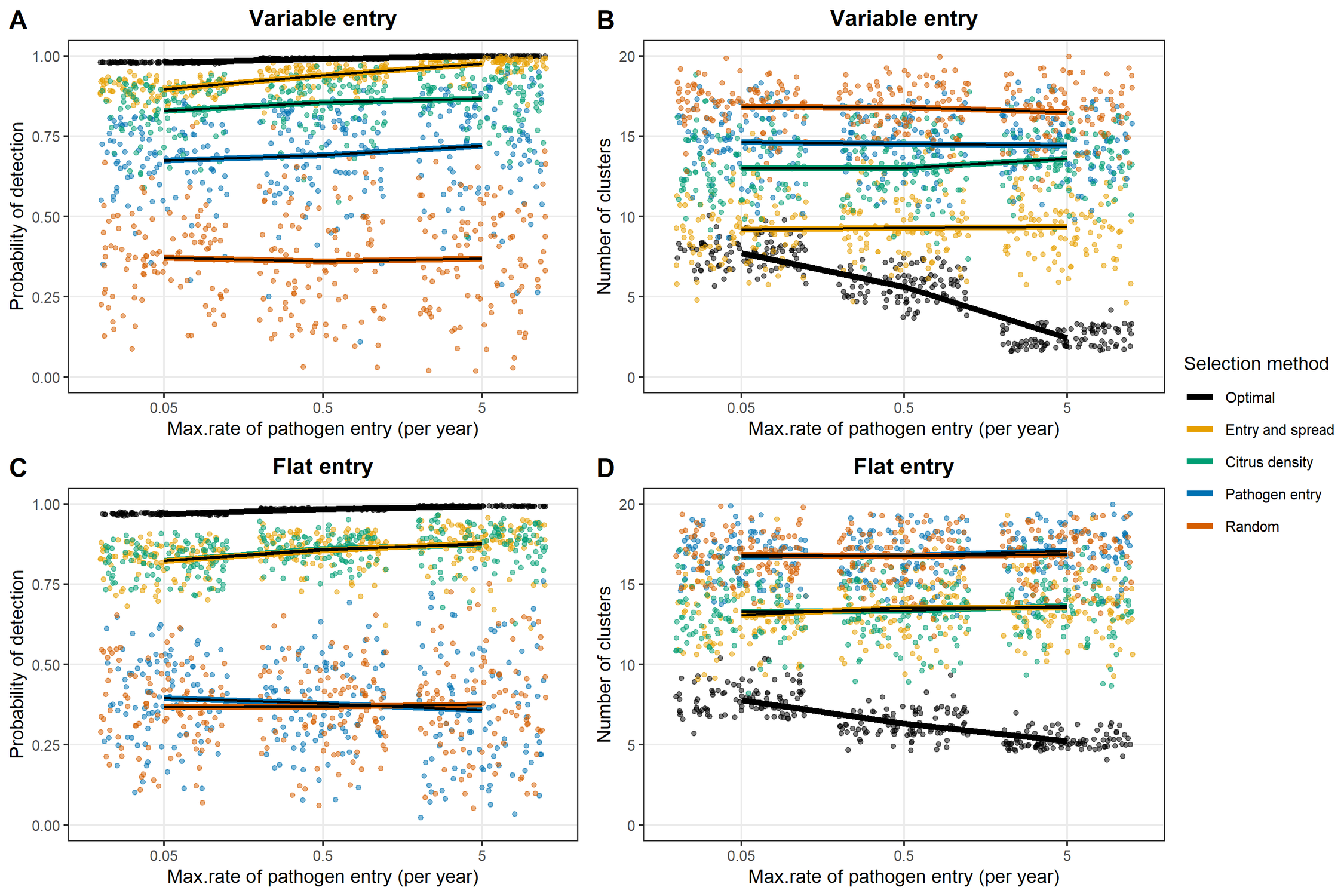

Supplement: S3 Fig — These plots show the impact of rate of pathogen entry on the detection probability and number of clusters of sites within 20 km of each other for the different site-selection strategies explored in the manuscript. Plots A and B show the impact of varying the rate of pathogen entry on the overall mean probability of detection (A) and the total number of clusters (B) when the probability of pathogen entry into any given site is affected by the density of citrus host and the travel census probabilities. Plots C and D show the impact of varying the rate of pathogen entry on the overall mean probability of detection (C) and the total number of clusters (D) when only the citrus density influences the probability of pathogen entry. All estimates are taken from 100 realisations for both the optimised sites and sites selected using different risk metrics. These metrics are the product of travel census probabilities and citrus density (‘Entry and spread’), citrus density, probability of entry according to the travel census model (‘Pathogen entry’), and random selection from the landscape. The data used to create these plots can be found at https://doi.org/10.17866/rd.salford.12759929.v1 (file ‘optimisationOutputs_primaryInf.csv’). (TIF) [file pbio.3000863.s004.tif]

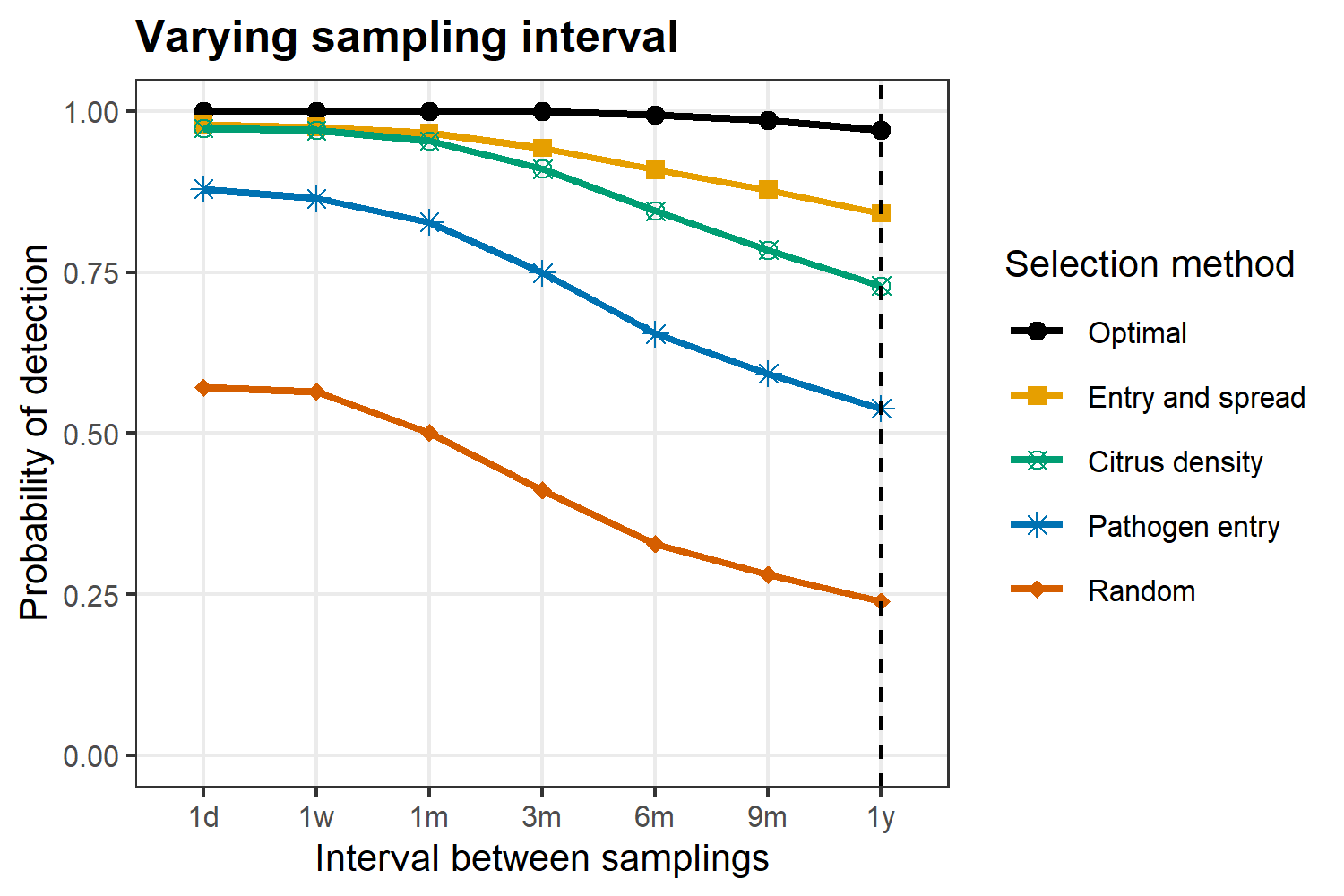

Supplement: S4 Fig — This plot shows the detection probability when the interval between sampling rounds is varied for both the optimised approach and a selection of different conventional targeted approaches using a variety of different metrics. All nonoptimised estimates are the mean of 1,000 sampling realisations in which the probability of site selection was based upon the site-specific measure of interest. These measures are the product of travel census probabilities and citrus density (‘Entry and spread’), citrus density, probability of entry according to the travel census model (‘Pathogen entry’), or random selection from the landscape. The optimised estimates are the mean of 10 optimisation runs. The dashed line indicates the ‘baseline’ scenario considered in the manuscript. The data used to create these plots can be found at https://doi.org/10.17866/rd.salford.12759929.v1 (file ‘samplingInterval.csv’). (TIF) [file pbio.3000863.s005.tif]

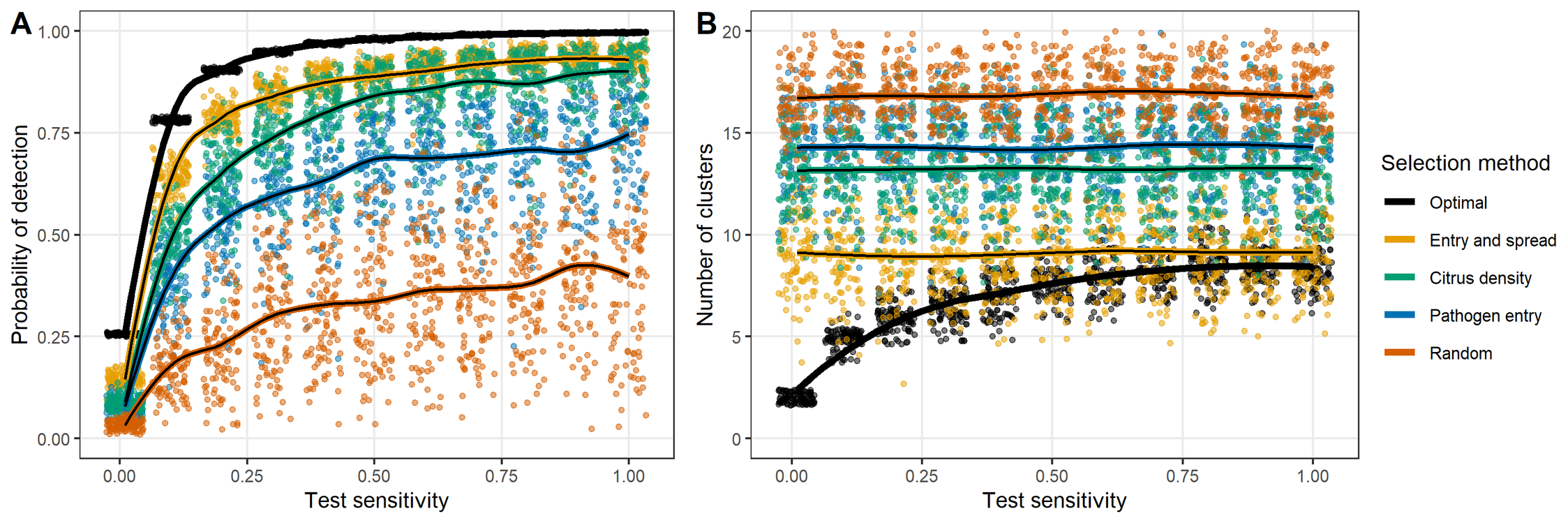

Supplement: S5 Fig — These plots show the effect of varying the test sensitivity and different site-selection strategies on the detection probability and number of clusters of selected sites. Each of 100 individual site selection runs are shown per selection strategy, along with the locally weighted regression curve. Plot A shows the impact on the probability of detection, and plot B shows the impact on the total number of clusters (with a cluster being all points within 20 km of each other). As well as the optimised strategy, sites were selected using 4 site-specific risk metrics: the product of travel census probabilities and citrus density (‘Entry and spread’), citrus density, probability of entry according to the travel census model (‘Pathogen entry’), and random selection from the landscape. The data used to create these plots can be found at https://doi.org/10.17866/rd.salford.12759929.v1 (file ‘optimisationOutputs_testSens.csv’). (TIF) [file pbio.3000863.s006.tif]

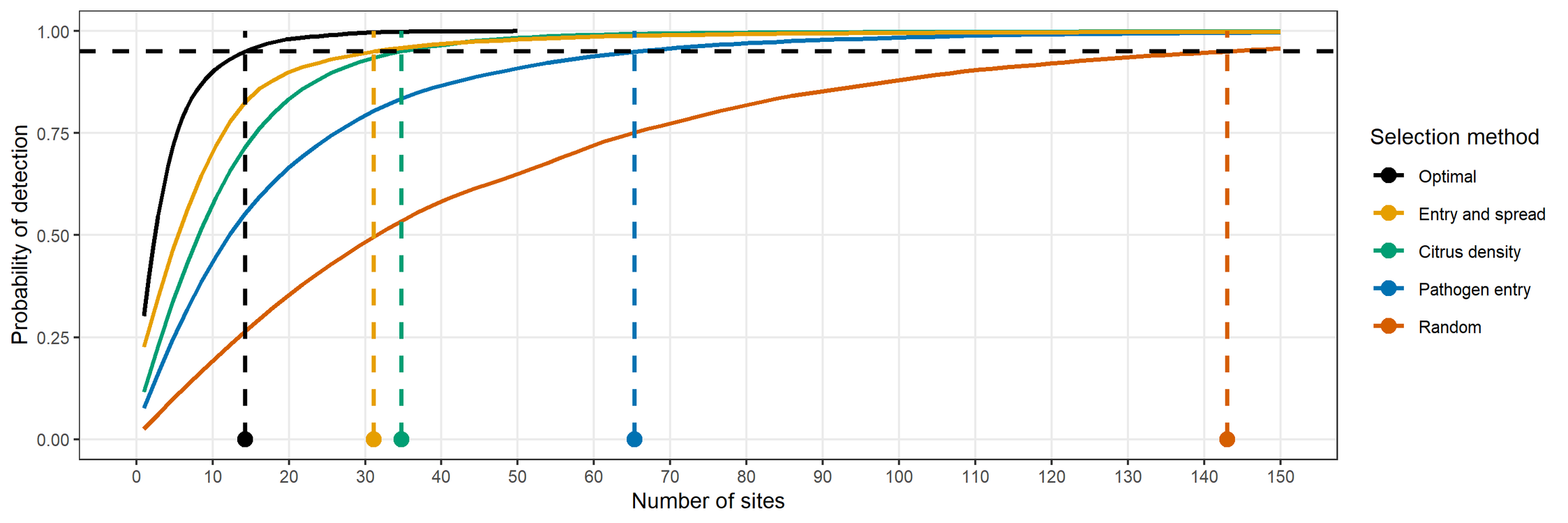

Supplement: S6 Fig — This plot shows the effect of varying the number of sites on the detection probability under a range of selection strategies. A total of 100 site selection runs were performed for each strategy and a locally weighted regression curve was fit to each. As well as the optimised strategy, sites were selected using 4 site-specific risk metrics: the product of travel census probabilities and citrus density (‘Entry and spread’), citrus density, probability of entry according to the travel census model (‘Pathogen entry’), and random (that is, unweighted) selection. Estimates of the probability of detection were made for all numbers of sites between 1 and 50 for all selection methods and additionally for all numbers of sites between 51 and 150 for the risk metric strategies. The horizontal dashed line shows a detection probability of 0.95, and the vertical dashed lines shows the mean number of sites required to achieve this detection probability under the different sampling strategies. The data used to create these plots can be found at https://doi.org/10.17866/rd.salford.12759929.v1 (file ‘optimisationOutputs_numSites.csv’). (TIF) [file pbio.3000863.s007.tif]

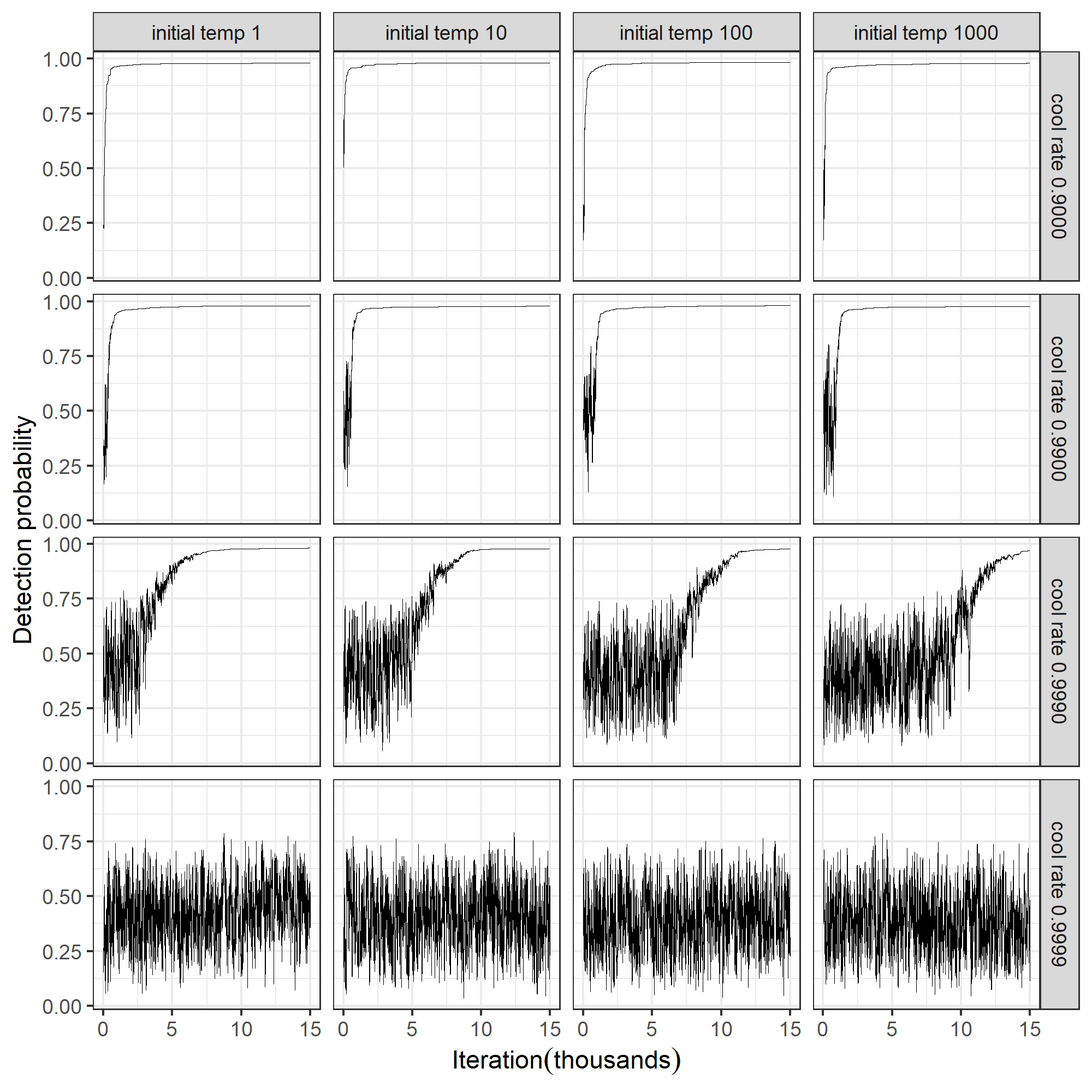

Supplement: S7 Fig — This plot shows the change the detection probability as the simulated annealing algorithm progresses over the first 15,000 iterations, using the baseline simulation and detection parameters. The final selected combination of initial temperature and cooling parameters were 10 and 0.9990, respectively (plot in the second column of the third row). The data used to create these plots can be found at https://doi.org/10.17866/rd.salford.12759929.v1 (file ‘ofPlots.csv’). (TIF) [file pbio.3000863.s008.tif]
