# Supplementary material for: Optimising risk-based surveillance for early detection of invasive plant pathogens
Source: PLoS Biol. 2020 Oct 12;18(10):e3000863. doi: 10.1371/journal.pbio.3000863 (PMC7581011; doi:10.1371/journal.pbio.3000863)
Supplement: Attachment — Submitted filename: Mastin_PBIOLOGY-S-20-00179_R1_response.docx [file pbio.3000863.s009.docx]

­­**Response to reviewers: Mastin et al. (PBIOLOGY-S-20-00179)**

**EDITOR’S COMMENTS**

You'll see that while all three reviewers are broadly positive about your study, they diverge regarding its readiness for publication in our journal. Only reviewer #2 is largely satisfied; reviewer #1 feels that the lack of biological insight drops it below the level required for PLOS Biology, and reviewer #3 is lacking an economic component.

Response: We thank the reviewers for these points, and we have made changes accordingly.

After discussion with the Academic Editor, we think that adding the extra aspect requested by reviewer #3 would strengthen your study significantly, and we would also recommend that you switch the paper to our "Methods and Resources article type, which does not have a formal requirement for novel biological insight, and which probably suits your study better overall.

a) When re-submitting, please select "Methods & Resources" as the article type.

Response: We appreciate your conclusion that our original submission was more suitable as a Methods and Resources Article. Our preference would be to submit as a Research Article, which we believe is reasonable now that we have better highlighted the fundamental science insights offered by our study. However, we are happy to submit as a Methods and Resources Article if you feel that this is still more fitting.

ACTION: This is done. We have resubmitted as a Methods and Resources Article, but would be grateful if you could consider our proposal above.

No re-formatting is required, though we appreciate reviewer #2's comment about bringing some methodological detail further forward in the paper (and we note that our "methods at the end" structure is not a hard-and-fast rule).

ACTION: This is done. We believe that the MS would be improved by including the Methods before the Results section, and so have made that change. We have also made the changes suggested by reviewer 2, which we believe improves the narrative even with this reorganisation. We are happy to return the Methods to the end of the MS if you prefer, however.

b) Reviewer #2 also requests that you make the data and code for this paper available. We'd like to emphasise that this is PLOS policy, and will be essential for publication.

ACTION. This is done. The simulation code, the optimisation code, and code for analysing the output of the optimisation have all been uploaded to github (<https://github.com/nikcunniffe/SpatialSampling>), and this has been noted in the MS (lines 181-186). Unfortunately, due to commercial sensitivities, we are unable to share the high-resolution citrus dataset, and therefore have provided a lower resolution version in order to allow this code to be run.

c) Regarding the request from reviewer #3 that you "add an economics layer," the Academic Editor says "The authors could be given a little more guidance on how to address the economic problem. If the method does indeed maximise the probability of detection before some specified threshold prevalence is reached, then it might be expected to be cost-effective relative to alternative methods. The authors could be asked to address the cost effectiveness question directly."

Response: Following a request for clarification, we received the following comments from the academic editor:

"Since the method they suggest involves the lowest number of sites needed to reach a given probability of detection it should also be cost-effective. If the cost per site is similar across methods it will absolutely do this.

"I don't see this as involving more than a couple of paragraphs, and a formal demonstration that, if cost per site is constant, the method that achieves a given probability of detection with the fewest number of sites is also the most cost effective."

ACTION: This is done. We are grateful for the reviewer for raising this point and for the subsequent clarification from the editors. We have included a new figure (Figure 5) and extra paragraphs to the text to address this, including the new “Surveillance costs” subsection of the Results (starting on line 373), and the “Capturing costs” subsection of the Discussion (starting on line 582).

Figure 5 shows how the additional number of sites required to achieve the same probability of detection differs across the selection methods. As the editor predicts, this demonstrates that the most cost-effective method, where costs per site are constant, is indeed given by the optimisation approach. Furthermore, it allows a direct comparison of the performance gain of the optimisation method, per unit cost, with the other methods. That is, the additional cost incurred to get to the same level of performance (detection probability) can be directly calculated. There is considerable practical merit to this since a method such as random sampling or sampling weighted by host density may be more readily implemented than the full optimisation approach, but this must be traded off against the extra investment needed to get to the same level of performance.

We add text to the results and to the discussion to highlight this and to explain how more elaborate economic costs could be captured (lines 594-620). The main aim of our current study is to consider surveillance in isolation of control. This allows us to identify what factors influence surveillance efficacy per se, before considering the impact of disease and control. We demonstrate that this is not trivial and depends on multiple interacting factors, including the sensitivity of the detection method. This new insight offered by our approach is now better communicated thanks to reviewer 1’s first comment which led to additional text (lines 463-475) and a new figure (Figure 7). To discuss how explicit economic issues could be built into our method we have rewritten the material in the “Surveillance aims” subsection of the Discussion as well as add additional text to highlight this as a direction for future work.

**REVIEWERS' COMMENTS:**

Reviewer #1:

This is a very interesting paper comparing the benefits of using a spatially explicit simulation model and optimisation to improve surveillance for achieving higher pest detection probabilities. The strongest aspect of this study is that the model/approach is operational, meaning it can give managers specific locations to target, which a lot of previous studies lack. However, I do not think the results are well enough explained or understood from a biological perspective. We can see that the optimisation routine improves detection probabilities quite dramatically, but why? The authors provide plausible explanations but because everything is numeric and related to a case study parameterisation, I'm left feeling that I don't understand why the optimisation is working so well. Just showing the optimisation works well because it is taking into account the whole problem is not enough, as there is a lot of similar case-studies that show this too. I'd expect more insights and a more interesting scientific story than "we developed a model, used an optimisation routine to improve detection, and it did well." Lots of papers tell that sort of story. For example in the abstract, there is no strong specific scientific insights described in the case study. It is a good, interesting paper, with some novel bits, and it is definitely publishable as is, in a more specialist journal. In my opinion though for a journal like PLOS Biology, I expect more biological insights.

Response: We thank the reviewer for these comments. We accept that we did not adequately communicate the scientific basis and new insight offered by our approach in the manuscript.

Our paper shows for the first time that the optimal arrangement of surveillance resources is influenced by the epidemiology of the pathogen and the characteristics of the detection method used to identify infection, and that these are not fully captured by convention methods of targeting surveillance. That is, we find that spatial correlation in risk can make it sub-optimal to focus solely on the highest risk sites, but that this depends on the interplay with other factors, such as the sensitivity of available detection methods.

We thank the author for their comments regarding the fact that our method is operational, but they are correct that we cannot reasonably consider the practical application of the method in isolation of the scientific basis upon which this rests (even if our method were to have purely practical value, decision makers would expect to understand why an optimised method outperforms more conventional strategies). As a result, we have expanded upon these aspects in the text and have including a dedicated figure summarising the value of optimisation.

ACTION: This is done. To explain the novel insight offered by our approach we have added a new schematic (Figure 7) in the Discussion, which demonstrates in a simple and intuitive way exactly why our optimised method outperforms conventional risk-based surveillance strategies. We have also added new text to explain this (abstract, Figure text, and lines 463-468 and 522-526). Through reference to the schematic example in Figure 7, we describe exactly how the two arms of our strategy (the simulation model and the optimisation algorithm) interact in order to identify the best arrangement of sites.

Some other comments

Ln 23 a common name or associated disease might be useful for non-specialists here

ACTION: This is done. We have clarified that the CODIRO strain of *X. fastidiosa* is “the cause of olive quick decline syndrome”, and that the agent responsible for wheat stem rust is *Puccinia graminis*.

Please state the biological description of the objective function, e.g. your goal is to select the spatial arrangement and sampling intensity at sites to maximize the probability of detecting the disease. There are a lot of possible objective functions one could choose, so really important to be explicit before getting into the methods at the end of the paper.

ACTION: This is done. We have clarified in the text (lines 83-84) that we are interested in maximising the probability of detecting new pathogen incursions before the threshold prevalence is reached.

Ln 336 - 360: I found this section especially strong. It relates to work on optimal surveillance in the invasive species/pest literature and why it's different from the approach considered here.

Response: We thank the editor for these comments. In response to comments from the other reviewers, the paragraph mentioned has been somewhat changed, but remains focused on the novelty of our method in comparison to previous work.

The fact that this approach is operational is a major advancement over past work. Some of this could be hinted at in the intro too if space allows [lines 42-50 seem like a natural place].

ACTION: This is done. We have added text to lines 75-76 which clarifies the operational nature of our method.

Ln 400 - 426: Flag what you are optimising over, is n fixed and just optimising over Omega? Also this info is not part of the optimisation "algorithm" it is part of the problem formulation. The optimisation algorithm is simulated annealing. So subtitles are a bit misleading.

Response: We thank the reviewer for pointing this out, and we can see how our ordering of subsections here was confusing.

ACTION: This is done. We have moved the ‘Optimisation algorithm’ subsection to before ‘Calculating the probability to detect at least one case of the epidemic’, and have renamed it ‘Optimisation approach’ (now starting on line 138). We therefore now first describe the optimisation strategy, before describing the technical details of how this was achieved, and have adapted the text accordingly.

Ln 374 - 462: Even after reading the full simulation and optimisation description, it is really unclear whether this is being thought of as a dynamic or static optimisation problem. Can the sampler change sites between rounds for example? My inclination is no. There are fixed spots where sampling occurs and the optimization statically evaluates where to sample once, and then continues to repeat sampling there. If my interpretation is incorrect and there is something in the supplement that spells everything out, that bit needs to be moved to the main text. Only selecting your sites at the first time step seems like a bit of a drawback as this can influence where a manager might choose at the start, if they cannot sample a new site later. If I have this wrong, I think a schematic diagram describing the order of events in the sampling and simulation might be useful.

Response: The reviewer is correct that our approach selects a static arrangement of sites. However, these sites are selected based upon the full dynamic (and stochastic) predictions of the spatial spread model. This method of focussing surveillance in specific locations is thus similar to ‘sentinel surveillance’. Whereas a dynamic selection strategy would have potential value, it would make it more difficult to answer the questions described in lines 86-95.

ACTION: This is clarified. We have added a clarifying statement to the end of the ‘Model-informed surveillance’ subsection in the discussion (lines 449-455), which explains that we consider a static arrangement of sites, and why we made this decision, but mentions the potential practical value of an adaptive strategy. We have also added a schematic diagram (Figure 7), which clearly demonstrates the basis of our approach.

Reviewer #2:

The authors propose an original approach for early detection of emerging infectious diseases of plants. This approach is grounded on a spatially explicit model of pathogen entry and spread, a statistical model of detection and a stochastic optimisation algorithm (simulated annealing). It is compared to several alternative surveillance strategies, including risk-based strategies, under several epidemiological and detection scenarios, and is applied in a real context, namely the emergence of huanglongbing in Florida, USA.

This manuscript describes a very good piece of work that deserves to be published.

Response: We thank the reviewer for their comments.

* The manuscript and the SI are well written even if the PLOS typical structure (intro - results - discussion - M&M) does not facilitate the reading of such a methodological work. I do not request changing the order of the sections, but I think that locating l.197-208 (slightly rephrased) before the initial presentation of the Figures at the beginning of the Results section would be more convenient and ease the understanding of the figures.

Response: We agree with the reviewer that the previous ordering of this text in relation to the figures was confusing.

ACTION: This is done. We have decided to move the Methods section to before the Results, but we felt that the suggested changes improved the flow of the MS even with this change, and have therefore made them as suggested (now starting on line 249). The text has also been slightly reworded so as to better explain the basis of the comparisons made.

* L.64-67 : This statement is, from my point of view, exaggerated. The authors present a definitely original work. However, anterior studies targeted quite close objectives. See e.g.:

- Colman E, Holme P, Sayama H, Gershenson C (2019) Efficient sentinel surveillance strategies for preventing epidemics on networks. PLOS Computational Biology 15(11): e1007517.

- P. Holme (2018). Objective measures for sentinel surveillance in network epidemiology. Phys. Rev. E 98, 022313

- Herrera, J., R. Srinivasan, J. Brownstein, A. Galvani, and L. A. Meyers (2016). Disease surveillance on complex social networks. 469 PLoS Computational Biology (12), e1004928.

- Martinetti D., Soubeyrand S. (2019). Identifying lookouts for epidemio-surveillance: application to the emergence of Xylella fastidiosa in France. Phytopathology 109: 265-276. I suggest to the authors to re-word the sentence by writing that they propose an original approach for addressing the question l.65-67.

Response: We thank the reviewer for pointing this out, and we do agree that our phrasing here was inadequate – ignoring as it does the considerable volume of work on surveillance targeting in networks. Indeed, these approaches are conceptually similar to our own in some ways.

ACTION: This is done. The text in lines 65-71 has been changed to more explicitly detail relevant work on network models, and to describe how our approach differs.

* About the number of repetitions for the optimal strategy. In Figures 3, 4, 5 and S2, the number of repetitions ranges from 1 to 10 for the optimal strategy whereas it ranges from 100 to 1000 for the other strategies. I guess performing the optimal strategy is particularly time-consuming and this explains the use of a reduced number of repetitions (by the way, can the authors provide typical computer time required?).

Response: Whilst the simulation and optimisation run very quickly, the checking and processing of the output needed for the current study can be quite time-intensive. Added to this is the large number of different scenarios compared throughout the MS.

ACTION: This is done. We have added text to state the running time for both the simulation and the optimisation in a new section of the Methods, on lines 187-193. Note that a bug in Word which we have not been able to correct means that this section is removed if all changes in the tracked changes version of the MS are accepted. The ‘clean’ version includes the section pasted back in manually.

However, the ms would be more convincing with larger numbers of repetitions, especially for providing evidence 1) that a low diagnostic sensitivity or a high rate of pathogen entry leads to a few survey clusters, and 2) that the advantage of the optimal strategy is still significant under incorrect assumptions, for which one has only a single repetition.

Response: The reviewer is correct that time is a major factor which precluded the repeated running of optimisations, especially when considering the large number of sensitivity analyses conducted in the current study. However, we appreciate that information on the variability of the performance of the optimised strategy is an important consideration for readers, as well as for the two points raised by the reviewer (number of clusters and robustness to spread assumptions).

ACTION: This is done. We have run 100 optimisations for the following scenarios:

- Impact of test sensitivity (11 different parameter values)
- Impact of rate of pathogen entry (three parameter values for each entry distribution pattern)

From these 1,700 optimisations, we demonstrate that the objective function (detection probability) is remarkably consistent (see new Figure 4) – thereby validating the general approach – and show that this remains consistent even in the face of model misspecification (see new Figure 6). We are also able to show the variability in the number of clusters. Although this is greater than for the detection probability, the general trends we discuss in the text remain clear.

We present these results in updated versions of Figures 4 and 5 (now Figures 4 and 6). We have also created two new supplementary figures (S3 and S5) to show the impact of varying the rate of pathogen entry and test sensitivity of the variability in the detection probability and the number of clusters between individual optimisations. As a result of these changes, the plot showing the effect of varying the sampling interval (previously in Figure 3) has been moved to the Supplementary Material (now Figure S4).

* L.165: (C) should be replaced by (D) and (D) by (E), shouldn't they?

* L.234: Figure S2 instead of Figure S3, isn't it?

Response: We thank the reviewer for noticing these errors.

ACTION: This is done. These figures have now been changed, and the MS has been checked for any other similar errors.

* L.354-357: [47] uses a network-based formalization indeed, but the nodes of the network actually correspond to the cells of a grid covering a continuous real landscape, as in the authors' work.

Moreover, the following articles dealing with the search of improved surveillance strategies for Sharka with spatially-explicit stochastic epidemic/observation models are closely related to the points discussed in the last paragraph of the discussion and could also be cited:

- Picard C., Soubeyrand S., Jacquot E., Thébaud G. (2019). Analyzing the Influence of Landscape Aggregation on Disease Spread to Improve Management Strategies. Phytopathology, PHYTO-05.

- Rimbaud L., Dallot S., Bruchou C., Thoyer S., Jacquot E., Soubeyrand S., Thébaud G. (2019). Improving management strategies of plant diseases using sequential sensitivity analyses. Phytopathology 109: 1184-1197.

Response: We thank the reviewer for bringing these studies to our attention. Indeed, these do relate to the current approach, as well as to our expanded discussion of the consideration of control costs, as requested by reviewer 3.

ACTION: This is done. We have reformulated the “Surveillance aims” subsection of the Discussion in order to discuss these studies (as well as the network studies suggested earlier), and demonstrate both the similarities and differences with our work. These are now captured in the “Capturing costs” subsection (starting on line 582).

* For reproducibility reasons, for allowing the application of the authors' approach to other case studies, but also for allowing methodological work on the comparison of different optimization approaches for early detection, in the vein of the authors' proposal, it would be relevant to provide the data, the code of the simulator and the code of the surveillance strategies on referenced open-access archives.

Response: We fully agree that this is essential, and we apologise for not providing these at original submission.

ACTION: This is done. We have uploaded the following code to github (https://github.com/nikcunniffe/SpatialSampling):

- Simulation code in C (with config file)
- Optimisation code in C (with config file)
- Code to generate some simple georeferenced figures from the output.

We have also added text in lines 181-186 to point the reader towards this. Note that a bug in Word which we have not been able to correct means that this section is removed if all changes in the tracked changes version of the MS are accepted. The ‘clean’ version includes the section pasted back in manually.

Reviewer #3:

A spatially explicit model of disease spread is developed and applied to the economically damaging huanglongbing disease, to identify the arrangement of surveillance sites that maximise the probability of early detection. The authors are interested in understanding whether surveillance resources should be placed around a single high-risk site or spread across other potential introduction sites as well. Mainland Florida is used as the case study application.

Given that the problem addressed in this paper is one of optimal resource allocation, my comments and concerns focus on the absence of any economic variables in the model. This is despite the importance of economic factors being alluded to: e.g. (line 38) "… an effective early detection strategy is complicated by … limited financial and logistical resources."; (lines 33-34) "the 'maximum accepted prevalence' will be impacted by … likely impact, and the availability and feasibility of control measures."; and (line 49-50) "there is a risk that the surveillance strategy may not be optimally targeted; resulting in … excessive costs"; (line 221) "… demonstrating the value of targeted surveillance strategies …".

Response: We thank the reviewer for raising these important points. We will respond in detail to the more specific points made below.

So, despite discussion about the importance of considering the performance of the detection method and spatial arrangement, we have no information about the economically optimal strategy. There is no indication of the various costs involved in their surveillance strategies - the assumption is therefore an unlimited budget.

ACTION: This is done. Following discussion with the editors, we have conducted further analyses which estimate the surveillance costs explicitly, and discuss the impact of the different site selection strategies on these costs.

Pest managers, who regularly decide how to spend limited resources on disease control, would require information on the costs and trade-offs involved in various surveillance strategies in order to make these complicated resource allocation decisions. In essence, to obtain the greatest benefits from investing scarce resources, surveillance design must explicitly balance the cost of surveillance with the costs of allowing invading populations to go undetected longer (Epanchin-Neill et al., 2014).

In my view, therefore, the model is incomplete -an economics layer needs to be included in the model and the analysis before the article can be considered for publishing. I leave this to the authors to decide on the best way of including the economics.

Response: As we discuss in the original manuscript (and expand upon in the resubmitted version), we agree that control costs are of great importance, but are not the main focus of our current work. By focussing for the first time on detection before a threshold prevalence, we are able to identify important factors which influence surveillance efficacy in isolation of the control costs. As the importance of surveillance is increasingly recognised, we believe that this is an important and timely issue worthy of investigation in its own right.

ACTION: This is done. We are grateful to the reviewer for highlighting this important point. The editors provided further comment on how to address this. This was clarified further to us in an exchange of emails with the editors. Please see cover letter page 1 for our response, and the new “Surveillance costs” subsection of the Results (starting on line 373), and the “Capturing costs” subsection of the Discussion (starting on line 582) for a more comprehensive discussion of the inclusion of economic factors.

Note that while some of the important literature on early detection that includes both biology and economics is cited, the authors appear to be critical of these papers in their focus on the optimal balance of early detection surveillance and control intensity required to minimise costs (lines 61-63 and 348-353). Others articles worth reviewing are Kean and Stringer (2019); and Epanchin-Neill and Hastings, 2010).

Linked to the above, the authors claim that no previous studies have addressed the question of where surveillance resources should be located to maximise the probability of detecting an invading pathogen before it reaches a certain prevalence. I would suggest this is because in those previous studies the authors have chosen to solve the much more relevant and pressing problem of developing surveillance strategies that balance the cost and benefits involved in particular designs

Response: We accept that, in our attempt to demonstrate the novelty of our current approach, we may have appeared to be overly critical of these studies in the original submission. This was not our intention.

ACTION: This is done. We have reworded the text in the Discussion in order to better clarify where our study is positioned in relation to these studies (and to other studies using network analysis methods to target surveillance, as suggested by reviewer 2). These are detailed in lines 65-71 and lines 583-594.

Line 33: 'maximum acceptable prevalence': how is this determined, and could it based on balancing the cost of surveillance with the costs of allowing invading populations to go undetected longer?

Response: The reviewer is exactly right that this would be one way of specifying this prevalence threshold, and one which could be operable within the current framework.

ACTION: This is done. We have added text on lines 594-598 to describe in more detail how economic factors could be considered when specifying the threshold prevalence, and how this can link in with conventional strategies for regulatory surveillance.

Line 78-79: "How easy is it to implement this new strategy" - what does 'easy' mean in this context and how will it be evaluated?

Response: We appreciate that this point is unclear, and we are very grateful for the reviewer raising it. Our intention was to consider here how widespread the optimal surveillance effort was, but we appreciate that we did not quantify this during our analysis, beyond the cluster calculations.

ACTION: This is done. We have removed this portion of text.

Line 86: what is meant by 'general surveillance'?

Response: Here, we were interested in identifying “general surveillance designs” which can approximate the optimised one. That is, whether the optimised method can be easily replicated or approximated without formal optimisation. We largely focused on the value of risk-based strategies in the current study, but we appreciate that we did not make this point very clear.

ACTION: This is done. We have added text to the original question (line 93) to clarify our meaning, and have expanded upon the text in lines 471-473 of the Discussion to more explicitly answer the question.

Line 189-190: the model uses a pre-defined 'threshold prevalence' of 1% to indicate the point at which control would no longer be possible. This seems like an arbitrary value, and some additional explanation is required. For example, has the value of control at a prevalence of 1% been estimated? What is 'control'?

Response: This threshold was selected to represent the highest prevalence at which control could still be feasible. However, we appreciate that this was not made explicit in the manuscript. The cost of control at this prevalence has not been explicitly estimated, but it would be valuable to do so if the method was being applied contemporarily.

ACTION: This is done. We have added text to lines 348-351 to describe the arbitrary nature of the threshold, but also to clarify that it could alternatively be selected based on regulatory guidance and/or economics. We also briefly describe one method of considering control costs when specifying the threshold prevalence in the new “Capturing Costs” subsection of the Discussion (lines 594-598).

Table 1: 'pathogen entry', could additional explanation be provided on how entry is 'seeded' - is it a random process in a particular cell?

ACTION: This is done. We have added text to the Supplementary Information describing this process.

In summary, adding an economic component to the model would make it a very publishable paper.

ACTION: This is done, as described above.

References

Bradhurst, R. A., et al. (2015). "A hybrid modeling approach to simulating foot-and-mouth disease outbreaks in Australian livestock." Frontiers in Environmental Science 3(17).
Epanchin-Niell, R. S. and A. Hastings (2010). "Controlling established invaders: integrating economics and spread dynamics to determine optimal management." Ecology Letters 13(4): 528-541.
Epanchin-Niell, R. S., et al. (2014). "Designing cost-efficient surveillance for early detection and control of multiple biological invaders." Ecological Applications 24(6): 1258-1274.
Kean, J. M. and L. D. Stringer (2019). "Optimising the seasonal deployment of surveillance traps for detection of incipient pest invasions." Crop Protection 123: 36-44
