# Supplementary material for: Optimising risk-based surveillance for early detection of invasive plant pathogens
Source: PLoS Biol. 2020 Oct 12;18(10):e3000863. doi: 10.1371/journal.pbio.3000863 (PMC7581011; doi:10.1371/journal.pbio.3000863)
Supplement: Attachment — Submitted filename: Mastin_PBIOLOGY-D-20-00233R2_response.docx [file pbio.3000863.s010.docx]

­­**Response to reviewers: Mastin et al. (PBIOLOGY-D-20-00233R2)**

**EDITOR’S COMMENTS**

Note that we do not require all raw data. Rather, we ask that all individual quantitative observations that underlie the data summarized in the figures and results of your paper be made available in one of the following forms:

1) Supplementary files (e.g., excel). Please ensure that all data files are uploaded as 'Supporting Information' and are invariably referred to (in the manuscript, figure legends, and the Description field when uploading your files) using the following format verbatim: S1 Data, S2 Data, etc. Multiple panels of a single or even several figures can be included as multiple sheets in one excel file that is saved using exactly the following convention: S1_Data.xlsx (using an underscore).

2) Deposition in a publicly available repository. Please also provide the accession code or a reviewer link so that we may view your data before publication.

Regardless of the method selected, please ensure that you provide the individual numerical values that underlie the summary data displayed in all main and Suppementary Figure panels as they are essential for readers to assess your analysis and to reproduce it.

Please also ensure that figure legends in your manuscript include information on where the underlying data can be found, and ensure your supplemental data file/s has a legend.

Please ensure that your Data Statement in the submission system accurately describes where your data can be found.

ACTION: We have added all of the datasets required to recreate the plots to the University of Salford’s figshare repository (DOI 10.17866/rd.salford.12759929), along with a readme file detailing the contents of each dataset. The datasets are each linked to explicitly in the Figure legends.

**REVIEWERS' COMMENTS:**

Reviewer #1:

The authors have mostly addressed all my comments adequately. The abstract is especially more insightful, adding the "correlation" component, I can now better understand why its working. I think the new format is also beneficial. My only issue is that it is not explicitly stated in the optimisation summary whether n is fixed beforehand, or whether sets of sites that vary in size are considered by the optimization routine. I assume the latter, but the text makes it seem like the former, but doesn't explicitly say so.

Response: We thank the reviewer for pointing out this oversight.

ACTION: We have added text to lines 116-117 stating that the number of samples per site is fixed.

Reviewer #3:

This paper, as suggested in the abstract (line 5) is about "how best to target surveillance resources to achieve [detection of epidemics at an early stage]". This is therefore a problem about optimal resource allocation, thus the costs and benefits of control must be considered properly. I acknowledge that the discussion of the economics aspects of the problem has improved - e.g. control costs have been included -but the benefits of control (damages/impact avoided because control is applied) are also an important consideration and are not fully discussed. There is a trade-off between spending on surveillance for HLB and the cost of controlling future spread which would otherwise cause damage. This trade-off will also be important in choosing the 'threshold prevalence', but is still absent from the discussion.

When considering threshold prevalence the authors acknowledged that this represents 'the highest prevalence at which control could still be feasible'. The term 'feasible' is key, and again, has economic connotations that can't be ignored. Feasibility includes the costs of control and the benefits of control - the damages that are avoided because of control. Benefits should be incorporated into the discussion.

Other comments have been dealt with appropriately.

Response: We thank the reviewer for raising these important points. Although costs and benefits are not the main focus of the work, they underpin the practical implications of our approach, and we appreciate that we have not yet explicitly discussed surveillance benefits sufficiently. As the reviewer suggests, we have now added these to the discussion.

ACTION: We have added new text to the start of the ‘Capturing Costs’ subsection of the Discussion (lines 593-598), explaining that the detection probability itself (that is, the objective function over which we optimise) does not itself capture the costs and the benefits of the surveillance scheme. In lines 609-615, we then explain that our main focus is not economic optimisation and list the costs which would need to be considered if this was the case. Finally, in lines 611-619, we explain how our method could incorporate these costs and describe a practical approach which could be applied whilst specifying the ‘threshold prevalence’, as suggested by the reviewer. Note that in lines 609-619, we do not describe the benefits of surveillance as such, but rather consider them as the expected costs in the absence of a surveillance scheme (which we compare to the expected costs in the presence of a surveillance scheme). We also now explicitly mention benefits as well as costs in the description of further work in line 652.

We believe the new insight our approach offers, into how the interplay between epidemiology and detection method influence the choice of optimal surveillance locations, will help shed light on approaches which rely on detailed/complex economic optimization.
